# Quinolone Resistance in Absence of Selective Pressure: The Experience of a Very Remote Community in the Amazon Forest

**DOI:** 10.1371/journal.pntd.0001790

**Published:** 2012-08-28

**Authors:** Lucia Pallecchi, Alessandro Bartoloni, Eleonora Riccobono, Connie Fernandez, Antonia Mantella, Donata Magnelli, Dario Mannini, Marianne Strohmeyer, Filippo Bartalesi, Hugo Rodriguez, Eduardo Gotuzzo, Gian Maria Rossolini

**Affiliations:** 1 Dipartimento di Biotecnologie, Sezione di Microbiologia, Università di Siena, Siena, Italy; 2 Dipartimento Area Critica Medico Chirurgica, Clinica Malattie Infettive, Università di Firenze, Florence, Italy; 3 Malattie Infettive e Tropicali, Azienda Ospedaliero-Universitaria Careggi, Florence, Italy; 4 Hospital Apoyo Yurimaguas, Yurimaguas, Peru; 5 Instituto de Medicina Tropical Alexander von Humboldt, Universidad Peruana Cayetano Heredia, Lima, Peru; 6 Dipartimento di Emergenza, Urgenza e dei Servizi Diagnostici, U. O. Microbiologia e Virologia, Azienda Ospedaliera-Universitaria Senese, Siena, Italy; Universidad Peruana Cayetano Heredia, Peru

## Abstract

**Background:**

Quinolones are potent broad-spectrum bactericidal agents increasingly employed also in resource-limited countries. Resistance to quinolones is an increasing problem, known to be strongly associated with quinolone exposure. We report on the emergence of quinolone resistance in a very remote community in the Amazon forest, where quinolones have never been used and quinolone resistance was absent in 2002.

**Methods:**

The community exhibited a considerable level of geographical isolation, limited contact with the exterior and minimal antibiotic use (not including quinolones). In December 2009, fecal carriage of antibiotic resistant *Escherichia coli* was investigated in 120 of the 140 inhabitants, and in 48 animals reared in the community. All fluoroquinolone-resistant isolates were genotyped and characterized for the mechanisms of plasmid- and chromosomal-mediated quinolone resistance.

**Principal Findings:**

Despite the characteristics of the community remained substantially unchanged during the period 2002–2009, carriage of quinolone-resistant *E. coli* was found to be common in 2009 both in humans (45% nalidixic acid, 14% ciprofloxacin) and animals (54% nalidixic acid, 23% ciprofloxacin). Ciprofloxacin-resistant isolates of human and animal origin showed multidrug resistance phenotypes, a high level of genetic heterogeneity, and a combination of GyrA (Ser83Leu and Asp87Asn) and ParC (Ser80Ile) substitutions commonly observed in fluoroquinolone-resistant clinical isolates of *E. coli*.

**Conclusions:**

Remoteness and absence of antibiotic selective pressure did not protect the community from the remarkable emergence of quinolone resistance in *E. coli*. Introduction of the resistant strains from antibiotic-exposed settings is the most likely source, while persistence and dissemination in the absence of quinolone exposure is likely mostly related with poor sanitation. Interventions aimed at reducing the spreading of resistant isolates (by improving sanitation and water/food safety) are urgently needed to preserve the efficacy of quinolones in resource-limited countries, as control strategies based only on antibiotic restriction policies are unlikely to succeed in those settings.

## Author Summary

Quinolones are broad-spectrum antibiotics which bind to type II topoisomerases (DNA gyrase and topoisomerase IV) and inhibit DNA re-ligation after enzyme cut, exerting a rapid bactericidal activity. They are widely used for the treatment of several community- and hospital-acquired infections and have become increasingly important also in resource-limited countries, following the availability of generics (which have drastically reduced drug costs) and the remarkable increase of resistance to the oldest and cheapest antibiotic classes. Resistance to quinolones is an increasing worldwide problem that challenges the efficacy of these drugs against several bacterial pathogens and is known to be strongly associated with quinolone exposure. Restriction of quinolone consumption has been advocated as an important tool for the containment of quinolone resistance and has recently been proved to succeed in reducing resistance rates in clinical isolates of *Escherichia coli* in a community setting from an industrialized country. This study describes the dissemination of quinolone resistant *E. coli* in a very remote community in the Amazon forest, with a high level isolation and minimal antibiotic use, not including quinolones. These findings demonstrate that intervention strategies based only on quinolone restriction are unlikely to succeed in resource-limited countries, unless accompanied by measures for reducing dissemination of resistant isolates by improving sanitation.

## Introduction

Quinolones are broad-spectrum antimicrobial agents with rapid bactericidal activity, overall low toxicity, and the possibility of being administered either orally or parenterally. Thanks to these features, quinolones are drugs of choice for the treatment of several community- and hospital-acquired infections (such as respiratory tract infections, skin and soft-tissue infections, urinary tract infections, gastro-intestinal infections, gonorrhea, tuberculosis, etc.), being among the most prescribed antibiotics [1]. Moreover, despite pediatric use has been restricted due to concerns with bone cartilage toxicity, quinolones are increasingly prescribed also for the treatment of life-threatening infections in pediatric patients [2], [3].

Following the broad dissemination of pathogens with acquired resistance to the older and less expensive antibiotics (e. g. ampicillin, tetracycline and trimethoprim-sulfamethoxazole), and the recent release of low-cost generic ciprofloxacin, the consumption of quinolones has significantly increased also in resource-limited countries [4], [5]. In those settings, where the newest and patent-protected antimicrobial compounds are not easily available, quinolones have become key drugs for the treatment of common bacterial infections, including those with a major impact in morbidity and mortality, such as dysentery and typhoid fever [6], [7].

As observed with all other antimicrobial agents, also the quinolones are affected by bacterial resistance. Acquired quinolone resistance has been reported among all major bacterial pathogens, and has attained very high level rates in several important Gram-positive and Gram-negative pathogens (including *Staphylococcus aureus*, *Neisseria gonorrhoeae*, *Escherichia coli*, *Klebsiella pneumoniae*, *Salmonella enterica*, *Shigella* spp., *Pseudomonas aeruginosa, Acinetobacter* spp., *Helicobacter pylori*) in some settings [1], [4]–[6], [8]–[10].

Quinolone resistance generally arises in a stepwise manner, following chromosomal mutations that alter the topoisomerase targets or upregulate bacterial efflux systems. In *Enterobacteriaceae*, several plasmid-mediated resistance mechanisms to quinolones (PMQR) have also been detected, including the Qnr proteins (that protect the topoisomerase targets), the AAC-cr enzyme (that inactivates some quinolones by acetylation), and the QepA and OqxAB efflux systems (which are able to extrude some quinolones) [11]. Although these PMQR mechanisms are able to confer only low level resistance to quinolones, their presence is thought to facilitate the emergence of chromosomal mutations leading to resistance levels of clinical significance [11].

A clear relationship has been demonstrated between the emergence and dissemination of quinolone resistance among bacterial pathogens and fluoroquinolone use, both in hospital and community settings [5], [12]–[14], while a recent study has also reported a rapid decrease of quinolone resistance rates in clinical isolates of *E. coli* after a countrywide intervention of quinolone restriction [15]. The relationship between quinolone use and resistance has also been indirectly supported by the absence or very low rates of acquired resistance to these drugs in the few studies which investigated antibiotic susceptibility in enterobacteria isolated from humans or wild animals living in remote areas of the planet away from anthropogenic drug exposure [16], [17].

Here we report on the experience with a very remote community of the Peruvian Amazon forest, where quinolone resistance in commensal *E. coli* was found to be completely absent in 2002 [18], but present at remarkable rates in 2009, notwithstanding that during this period the community had retained a condition of high level of geographical isolation, limited exchanges with the exterior, minimal antibiotic exposure and absence of quinolone availability.

## Materials and Methods

### Ethics Statement

Full ethical clearance was obtained from the qualified local authorities who had revised and approved the study design and consent form (Comité Institucional de Ética de la Universidad Peruana Cayetano Heredia, Lima, Peru). Before the fieldwork started, representatives of the local healthcare authorities and the research team met the community leader and adults to explain the purpose and procedures of the survey.

All the inhabitants of the community were considered eligible for the study. Prior to their enrollment, written informed consent was obtained from all adult participants and from the parents or legal guardians of minors. Any literate participant signed the consent form. In case of an illiterate participant, the consent form was read and signed by a witness (who was present throughout the consent procedure and interview) and marked by the participant/parent. Consent procedure and interviews were always conducted by trained local healthcare workers with the help of a local translator.

### Study Design and Population

Angaiza is a community of Chayahuita ethnic group located in the Alto Amazonas province of Peru. It was selected by the local healthcare authorities as being one of the most isolated community of the Peruvian Amazonas. In fact, from the nearest urban area (Yurimaguas, about 32,000 inhabitants), Angaiza can be reached by a 13-hour trip, including a 2-hour drive on an unpaved road followed by a 4-hour motor boat ride and a final 7-hour walk in the jungle. The population lives in typical Amazon huts including a single room, without sanitation and electricity, and locally collected rainwater represents the only water source. The principal activities are agriculture, hunting and animal breeding (poultry, pigs and cows). Healthcare available consists of the visits of a professional healthcare worker approximately every 4 months, and primary care for the most common illnesses provided by a volunteer from the community.

A previous study on fecal carriage of antibiotic resistant enterobacteria among the inhabitants of Angaiza was performed in 2002 [18].

At the time of the 2009 survey, the Chayahuita community comprised 140 individuals, living in 21 households. One hundred twenty members of the community (86%) consented to participate in the study (female-to-male ratio 61∶59, age range 0–71 years, mean age 17 years, median age 12 years) (Table 1). Study participants were representative of all the 21 households of the community (mean and median study participants per household was 6 and 6, respectively).

**Table 1:** Characteristics of the study community: 2002 vs. 2009.

	2002	2009
**General characteristics of the community**		
Individuals living there	113	140
Households	15	21
Travel time from the nearest urban area (Yurimaguas)	13 hours^a^	unchanged
Use of water potentially contaminated by other human populations	excluded	unchanged
Visit by a professional healthcare worker	∼every 4 months	unchanged
Antimalarial drugs stored	yes^b^	none
Antibacterial drugs stored	none	yes^c^
Non-human antibiotic use	absent	unchanged
**Features of the study population**		
Individuals included in the study	89 (79%)	120 (86%)
Households	15	21
Male-to-female ratio	48∶41	61∶59
Age range (years)	0–59	0–71
Mean age (years)	14.9	17
Median age (years)	9	12
Total no. of travellers^d^	19 (21%)	33 (27%)
Households with at least one traveller	10 (67%)	15 (71%)
Individuals reporting previous antibiotic use^e^	3 (3%)	5 (4%)
Households with at least one member reporting antibiotic use	2 (13%)	3 (14%)
Antibiotics used (no. of individuals)^f^	TET (3)	AMP (3); SXT (2)

a 2-hour drive on a unpaved road, 4-hour motor boat ride, 7-hour walk in the jungle.

b Chloroquine and primaquine (introduced following a previous malaria epidemic).

c Ampicillin, dicloxacillin, erythromycin, and trimethoprim-sulfamethoxazole.

d Traveller, an individual with a history of travel to Yurimaguas (the nearest urban area) in the 12 months preceding the survey. No statistically significant difference between 2002 and 2009 (*P*>0.37).

e Individuals reporting antibiotic use in the two weeks preceding the survey. No statistically significant difference between 2002 and 2009 (*P* = 0.76).

f TET, tetracycline; AMP, ampicillin; SXT, trimethoprim-sulfamethoxazole.

The study was conducted during a two-day visit to the community (December 12–13, 2009). Specially prepared forms were used to collect data from the community leader (about the general characteristics and organization of the community) and from each individual/legal guardian of children included in the study (about travels outside the community and previous antibiotic use). For the microbiological investigation, a stool sample was collected from each individual who consented to participate in the study, and a fecal swab was obtained from each sample. Moreover, fecal swabs were obtained from 48 animals reared in the community, including poultry (n = 19), pigs (n = 13), dogs (n = 8), cattle (n = 6) and cats (n = 2). Fecal swabs were stored in Amies transport medium (Oxoid, Milan, Italy) and transferred within 48 hours to the laboratory of Santa Gema Hospital of Yurimaguas.

### Screening for Fecal Carriage of Antibiotic Resistant *E. coli*

Fecal carriage of antibiotic resistant *E. coli* was investigated by a direct plating method, as described previously [18]–[20]. Briefly, each fecal swab was spread onto a MacConkey Agar No. 3 plate (MCA) (Oxoid, Milan, Italy) to yield uniform growth, and antibiotic disks were directly placed onto the seeded plate. After incubation at 37°C for 12–14 hours, plates were inspected for coliform growth, and inhibition zone diameters were measured and interpreted according to the previously described breakpoints [19], [20]. Criteria for differentiating between dominant and subdominant resistant population were the same as in the previous survey [19]. Briefly, a growth inhibition zone absent or smaller than the breakpoint diameter was suggestive of the presence of a resistant dominant population, while isolated colonies growing inside a growth inhibition zone of any size were considered suggestive of the presence of a resistant subdominant population. Antibiotics tested included ampicillin, ceftriaxone, tetracycline, trimethoprim-sulfamethoxazole, chloramphenicol, streptomycin, kanamycin, gentamicin, amikacin, nalidixic acid, and ciprofloxacin (Oxoid).

### Characterization of Fluoroquinolone Resistant Isolates

All fecal samples positive for the presence of a coliform population resistant to ciprofloxacin (17 from humans and 11 from animals) were streaked onto MCA plates supplemented with 5 µg/ml ciprofloxacin (MCA-CIP). One bacterial isolate exhibiting the morphology typical of *E. coli* was collected from each plate and identified by the API20E system (bioMérieux, Marcy l'Étoile, France). Susceptibility testing was performed by the disk diffusion method according to Clinical and Laboratory Standards Institute (CLSI) [21], [22]. *E. coli* ATCC 25922 was used for quality control purposes.

Detection of PMQR genes (*qnrA*, *qnrB*, *qnrC*, *qnrD*, *qnrS*, *aac(6′)-Ib-cr*, *qepA*) was performed by PCR and sequencing, as described previously [23]. Sequence analysis of *gyrA* and *parC* was carried out as described previously [24]. Nucleotide sequences were determined on both strands of PCR amplification products at the Macrogen sequencing facility (Macrogen Inc., Seoul, Korea).

Genotyping of ciprofloxacin resistant isolates was performed by determination of the main phylogenetic groups (A, B1, B2, D) using the Clermont method [23], Random Amplification of Polymorphic DNA (RAPD) using the 1290 decamer [23], and Multi Locus Sequence Typing (MLST) using protocols and conditions described on the *E. coli* MLST website [http://mlst.ucc.ie/mlst/dbs/Ecoli/documents/primersColi_html].

### Statistical Analysis

Data entry and analysis were performed with the Epi Info software package version 2008 (Centers for Disease Control and Prevention, Atlanta, GA). Statistical differences were determined by the Chi-Squared test. Confidence intervals were calculated by Stata Software release 8.0 (StataCorp. 2003).

## Results

### Characteristics of the Studied Community

Data obtained by the community leader and participants interviews showed that the characteristics of Angaiza were overall comparable to those observed in a similar survey carried out in 2002 [18] (Table 1). In particular, no major changes of the population structure, habits and healthcare organization had occurred since 2002. The most important difference consisted in the introduction of a panel of antibacterial drugs to be stored in the community (absent in 2002), including ampicillin, dicloxacillin, erythromycin and trimethoprim-sulfamethoxazole. Moreover, antimalarial drugs were no longer stored in the community, differently from 2002 when they had been introduced following a previous malaria epidemic (40% of individuals included in the 2002 study had received chloroquine in the two weeks preceding the survey [unpublished]).

During the 12 months preceding the survey, 33 individuals (27.5%) from 15 households had travelled to Yurimaguas (the nearest urban area), revealing a similar mobility rate compared to that observed in 2002 (Table 1). As far as antibiotic use is concerned, in the two weeks preceding the survey antibiotics were administered to five children (age range 0–5 years) from three households, for the treatment of diarrheal diseases. In particular, three children had received ampicillin and two trimethoprim-sulfamethoxazole (both drugs stored in the community) (Table 1). Moreover, six individuals reported use of antibiotics in the 12 months preceding the survey (excluding the last two weeks), although the type of antibiotic could not be identified. Despite the availability of some antibacterial compounds in the community, antibiotic use was found to be overall comparable to that observed in 2002 (*P* = 0.76 and *P* = 0.19 for use in the 2 weeks or 12 months preceding the survey, respectively). The usage of antibiotics for veterinary, husbandry and agricultural practices, and the use of animal feed remained totally absent, as they were in 2002.

### Fecal Carriage of Antibiotic Resistant *E. coli*: Emergence of Quinolone Resistance

Of the 120 individuals included in the 2009 survey, 119 (99%) were found to carry antibiotic resistant *E. coli* as part of their intestinal microbiota (92% in the 2002 survey, *P* = 0.008).

Compared to the previous survey [18], the most relevant findings were the overall increase of resistance rates (statistically significant for ampicillin, trimethoprim-sulfamethoxazole, streptomycin and kanamycin), and the emergence of resistance to quinolones at remarkable rates (45% to nalidixic acid, 14% to ciprofloxacin), which was completely absent in 2002 (Table 2). Of note, quinolone resistant *E. coli* represented the dominant enterobacterial population in a considerable proportion of individuals (Table 2). Carriers of quinolone resistant *E. coli* were found in 20 of the 21 households of the community. No significant association was found between carriage of quinolone resistant isolates and age, gender, travels to Yurimaguas (or living in a household with at least one member reporting previous travels to Yurimaguas) or antibiotic consumption (or living in a household with at least one member reporting previous antibiotic use) (data not shown).

**Table 2:** Individuals carrying antibiotic resistant *E. coli* as part of their intestinal microbiota: 2002 vs. 2009.

	2002 (n = 89)		2009 (n = 120)		*P*-value
	Total % [CI]	Dominant % [CI]	Total % [CI]	Dominant % [CI]	Total (Dominant)
Tetracycline	87 [78–93]	40 [30–51]	93 [87–97]	48 [39–58]	NS (NS)
Ampicillin	75 [65–84]	30 [21–49]	99 [95–100]	94 [88–98]	<0.001 (<0.001)
SXT	69 [58–78]	27 [18–37]	99 [95–100]	82 [74–88]	<0.001 (<0.001)
Streptomycin	66 [55–76]	19 [12]–[29]	98 [93–99]	88 [80–93]	<0.001 (<0.001)
Chloramphenicol	53 [42–63]	18 [11]–[28]	61 [51–70]	14 [8]–[22]	NS (NS)
Kanamycin	6 [2]–[13]	0 [0–4]	23 [15–31]	9 [5]–[16]	<0.001 (0.003)
Gentamicin	1 [0–6]	1 [0–6]	7 [3]–[13]	1 [0–5]	NS (NS)
Amikacin	0 [0–4]	0 [0–4]	0 [0–3]	0 [0–3]	NA
Ceftriaxone	0 [0–4]	0 [0–4]	0 [0–3]	0 [0–3]	NA
Nalidixic acid	0 [0–4]	0 [0–4]	45 [36–54]	19 [13]–[27]	<0.001 (<0.001)
Ciprofloxacin	0 [0–4]	0 [0–4]	14 [8]–[22]	2 [0–6]	<0.001 (NS)

CI, Confidence Intervals; SXT, trimethoprim-sulfamethoxazole; NS, not significant (*P*>0.05); NA, not applicable. Data of the 2002 survey are from ref. 18.

Investigation of fecal carriage of antibiotic resistant *E. coli* in 48 animals reared in the community (including poultry, pigs, cattle, dogs and cats) showed resistance rates overall similar to that observed in humans (Table 3). Of note, resistance to quinolones was widespread in all studied animal species.

**Table 3:** Animals carrying antibiotic resistant *E. coli* as part of their intestinal microbiota.

	Total (n = 48)	Poultry (n = 19)	Pigs (n = 13)	Dogs (n = 8)	Cattle (n = 6)	Cats (n = 2)
	n (%, CI)	n	n	n	n	n
Tetracycline	42 (88, 75–94)	15	13	7	5	2
Ampicillin	42 (88, 75–94)	17	13	8	2	2
SXT	45 (94, 83–98)	17	13	8	5	2
Streptomycin	44 (92, 80–97)	17	12	7	6	2
Chloramphenicol	39 (81, 68–90)	13	13	8	3	2
Kanamycin	6 (13, 6–25)	2	1	3	0	0
Gentamicin	8 (17, 9–30)	5	1	0	2	0
Amikacin	0 (0)	0	0	0	0	0
Ceftriaxone	1 (2, 0–11)	0	0	1	0	0
Nalidixic acid	26 (54, 40–67)	7	10	4	4	1
Ciprofloxacin	11 (23, 13–37)	3	5	2	1	0

CI, Confidence Intervals; SXT, trimethoprim-sulfamethoxazole.

### Molecular Analysis of Fluoroquinolone Resistant Isolates of Human and Animal Origin

All 28 ciprofloxacin-resistant *E. coli* isolates (17 and 11 of human and animal origin, respectively) were investigated for susceptibility phenotype, genetic background, and mechanisms of plasmid- and chromosomal-mediated quinolone resistance.

Ciprofloxacin-resistant isolates were always resistant to nalidixic acid and usually showed a multidrug resistance phenotype (defined as resistance to >1 antibiotic class), which mostly included trimethoprim-sulfamethoxazole (89%), tetracycline (86%), ampicillin (79%), streptomycin (68%), and chloramphenicol (57%) (Table 4).

**Table 4:** Features of 28 ciprofloxacin resistant *E. coli* isolates of human and animal origin.

RAPD type (No of. isolates)	Phylogenetic group	Strain	Origin	Household	Resistance other than to quinolones	QRDR substitutions	
						GyrA	ParC
A (6)	A	A2	Human	H1	SXT/TET/AMP/S	S83L, D87N	S80I
		A3	Human	H1	SXT/TET/AMP/S	S83L, D87N	S80I
		A5	Human	H1	SXT/TET/AMP/S	S83L, D87N	S80I
		A6	Human	H1	SXT/TET/AMP/S	S83L, D87N	S80I
		A21	Human	H3	SXT/TET/AMP/S	S83L, D87N	S80I
		PE001	Dog	H17	SXT/TET/AMP/KAN/GEN/CRO	S83L, D87N	S80I
B (6)	A	C30	Pig	H10	SXT/TET/CHL	S83L, D87N	S80I
		PL003	Chicken	H17	SXT/TET/CHL	S83L, D87N	S80I
		C006	Pig	H17	SXT/TET/AMP/S/CHL	S83L, D87N	S80I
		A86	Human	H15	SXT/TET/AMP/S	S83L, D87N	S80I
		A71	Human	H12	SXT/TET/S/CHL	S83L, D87N	S80I
		A105	Human	H19	SXT/TET/S/CHL	S83L, D87N	S80I
C (2)	A	C021	Pig	H3	-	S83L, D87N	S80I
		PE020	Dog	H3	-	S83L, D87N	S80I
D (1)	A	A75	Human	H13	SXT/TET/AMP/S/GEN/CHL	S83L, D87N	S80I
E (1)	A	V045	Cow	Community	TET/AMP	S83L, D87N	S80I
F (1)	A	C040	Pig	H21	SXT/TET/AMP/S/KAN/CHL	S83L, D87N	S80I
G (6)	B1	A91	Human	H16	SXT/TET/AMP/S/GEN	S83L, D87N	S80I
		A4	Human	H1	SXT/TET/AMP/S/CHL	S83L, D87N	S80I
		A10	Human	H2	SXT/TET/AMP/S/CHL	S83L, D87N	S80I
		A63	Human	H10	SXT/TET/AMP/S/CHL	S83L, D87N	S80I
		C031	Pig	H10	SXT/TET/AMP/CHL	S83L, D87N	S80I
		PT034	Duck	H10	SXT/TET/AMP/CHL	S83L, D87N	S80I
H (1)	B1	A66	Human	H11	SXT/TET/AMP/S/CHL	S83L, D87N	S80I
I (1)	B1	A103	Human	H19	SXT/TET/AMP/KAN	S83L, D87N	S80I
L (1)	D	A101	Human	H18	SXT/AMP/S/GEN/CHL	S83L, D87N	S80I
M (1)	D	A13	Human	H2	SXT/TET/AMP/S/CHL	S83L, D87N	S80I
N (1)	D	PL039	Chicken	H8	SXT/AMP/S/GEN/CHL	S83L, D87Y	S80I, A108V

SXT, trimethoprim-sulfamethoxazole; TET, tetracycline; AMP, ampicillin; S, streptomycin; GEN, gentamicin; CHL, chloramphenicol; CRO, ceftriaxone.

An overall genetic heterogeneity was observed among isolates of either human or animal origin. In fact, they were found to belong to various phylogenetic groups (61% group A, 28% group B1, and 11% group D), and to 12 different RAPD types (Table 4). The three most prevalent RAPD types (type A, including 6 isolates from 3 families; type B, including 6 isolates from 5 families; type G, including 6 isolates from 4 families) were detected both in humans and animals and were assigned to ST617, ST10, and ST224, respectively.

Sequencing the QRDR regions showed the presence of a double substitution in GyrA (Ser83Leu and Asp87Asn) and a single substitution in ParC (Ser80Ile) in 27 ciprofloxacin resistant isolates, and of a double substitution both in GyrA (Ser83Leu and Asp87Tyr) and ParC (Ser80Ile and Ala108Val) in the remaining one (Table 4). None of the PMQR genes investigated was detected.

## Discussion

In a previous survey, conducted in 2002, acquired quinolone resistance was found to be absent in *E. coli* from the inhabitants of Angaiza, a very remote community of Chayahuita ethnic group located in the Peruvian Amazonas and characterized by a considerable level of geographical isolation, limited contacts with the exterior, and minimal antibiotic use, which did not include quinolones [18]. In this study we showed that, despite the characteristics of the community remained substantially unchanged over a 7-year period, in 2009 quinolone resistant isolates were common in Angaiza, with carriage of nalidixic acid and ciprofloxacin resistant *E. coli* observed in 45% and 14% of the studied individuals, respectively. Quinolone resistant *E. coli* were also found to be common, at rates similar to those observed in humans, among animals of different species reared in the community.

The genetic heterogeneity observed among ciprofloxacin resistant isolates of human and animal origin excluded that the emergence of quinolone resistance in Angaiza was the consequence of the occasional introduction into the community of a highly successful quinolone resistant clone, capable of spreading and persistence even in the absence of selective pressure. These findings rather supported the hypothesis of a consistent influx of resistant isolates into the community, despite remoteness and limited exchanges with the exterior, with their persistence and dissemination in the absence of quinolone exposure being favored by the conditions of poor sanitation confirmed by the finding of RAPD types shared by humans and animals of different households. The multidrug resistance phenotype expressed by most ciprofloxacin resistant isolates would be consistent with a provenance from urban areas where they are selected by antibiotic exposure. In fact, the substitutions in the QRDR regions of GyrA (Ser83Leu and Asp87Asn) and ParC (Ser80Ile) detected in the ciprofloxacin resistant isolates from Angaiza are known to be among the most common cause of acquired high level fluoroquinolone resistance in clinical isolates of *E. coli* worldwide [25].

An influx of resistant isolates from urban areas to the remote community was also hypothesized to explain the high resistance rates to the oldest antibiotics (i. e. tetracycline, ampicillin, trimethoprim-sulfamethoxazole, streptomycin and chloramphenicol) observed in Angaiza in the 2002 survey [18]. Evidences supporting this scenario were represented by the similarities of resistance patterns and resistance genes observed between Yurimaguas (the nearest urban area) and Angaiza. On that occasion, the lack of quinolone resistance in the remote community was thought to reflect the fact that quinolone resistance rates in Yurimaguas were lower than those to the oldest antibiotics. In this perspective, the emergence of quinolone resistance in Angaiza would be consistent with the dramatic increase of carriage of quinolone resistant *E. coli* observed among healthy children in Yurimaguas in the period 2002–2005 (27% vs. 54% and 16% vs. 31% for nalidixic acid and ciprofloxacin, respectively) [20], [26].

In a recent study conducted in very remote villages of rural Guyana not exposed to quinolones, the finding of ciprofloxacin resistant *E. coli* was putatively ascribed to the heavy exposure to chloroquine, an antimalarial drug that can select for topoisomerase mutations conferring resistance to quinolones [27]. This does not seem to be the case for the emergence of quinolone resistance in Angaiza, since this community is located in a malaria endemic area where chloroquine has been used for a long time (e. g. 40% of individuals included in the 2002 study had received chloroquine in the two weeks preceding the survey), whilst quinolone resistance emerged only in recent years. Moreover, data from the 2009 interviews excluded a recent malaria outbreak in Angaiza and consumption of chloroquine in the two weeks preceding the survey, ruling out the hypothesis that the differences observed between 2002 and 2009 could have been related to an increased exposure to this antimalarial drug.

table-4-captionFluoroquinolones have become increasingly important in the therapeutic armamentarium of resource-limited countries, following the availability of generics (which drastically reduced drug costs) and the dramatic increase of resistance to the oldest and cheapest antibiotic classes [4], [5]. These broad-spectrum, stable and orally administrable antibiotics have entered among the first- and second-line choices for the treatment of several common bacterial infections, including enteric, respiratory and urinary tract infections, sexually transmitted diseases, as well as serious systemic infections (e. g. typhoid fever, urinary sepsis, bacteremia in severe malnutrition) [1], [2], [5]–[7]. Due to the frequent unavailability of the newer and patent protected antibiotics, the dissemination of fluoroquinolone resistance in resource-limited countries has worrisome clinical implications.

Acquired quinolone resistance in enterobacteria has been clearly associated with the use of fluoroquinolones [5], [12]–[14], being absent or exceedingly rare in remote areas of the planet away from anthropogenic drug exposure [16], [17]. Recently, a countrywide intervention of quinolone restriction in Israel resulted in a rapid decrease of resistance rates in clinical isolates of *E. coli* [15], suggesting that maintenance of quinolone resistance in enterobacteria could be strongly dependent on drug exposure. Our study provided new insights into this phenomenon, as we demonstrated that quinolone resistant *E. coli* (likely selected in urban areas under quinolone selective pressure) were able to widely disseminate and persist even in very remote settings not exposed to antibiotics. These findings proved that maintenance of quinolone resistance in *E. coli* is not always depended on drug exposure, as also suggested by recent studies on fitness cost of quinolone resistance [28], [29], and emphasized the key role of the intestinal microbiota in the dissemination of such a clinically relevant antibiotic resistance.

Overall, the results from the present study underline the urgent need for interventions aimed at improving sanitation and water/food safety to address the phenomenon of antibiotic resistance in resource-limited countries. Indeed, unless dissemination of resistant isolates is contained, control strategies based only on antibiotic restriction policies are unlikely to succeed in those settings, especially for bacteria able to colonize the human gut.

## Supporting Information

Alternative Language Abstract S1 Spanish translation of the Author Summary.
(DOCX)
