# Supplementary material for: Quinolone Resistance in Absence of Selective Pressure: The Experience of a Very Remote Community in the Amazon Forest
Source: PLoS Negl Trop Dis. 2012 Aug 28;6(8):e1790. doi: 10.1371/journal.pntd.0001790 (PMC3429404; doi:10.1371/journal.pntd.0001790)
Supplement: Alternative Language Abstract S1 — Spanish translation of the Author Summary. (DOCX) [file pntd.0001790.s001.docx]

Las quinolonas son un grupo de antibióticos de amplio espectro que actúan en el ADN cromosómico bacteriano, uniéndose a las topoisomerasas II (ADN girasa y topoisomerasa IV) e inhibiendo su acción con efecto bactericida. Esos antibióticos se usan frecuentemente para el tratamiento de una gran variedad de infecciones, tanto en el medio hospitalario como en el ámbito extrahospitalario. El uso de quinolonas (sobre todo de las fluoroquinolonas) se ha ido ampliando también en países de recursos limitados, a raíz de la disponibilidad de medicamentos genéricos (que han reducido drásticamente los costos) y del notable aumento de la resistencia a las más antiguas y más baratas clases de antibióticos.

La resistencia a las quinolonas es un problema, creciente en todo el planeta, que afecta la eficacia de estos fármacos contra varios patógenos bacterianos y que está fuertemente asociado con el uso previo de quinolonas. Por eso, la adopción de medidas de restricción del consumo de quinolonas se reconoce como una herramienta importante para la contención de la resistencia y recientemente se ha demostrado tener éxito en la reducción de las tasas de resistencia en aislados clínicos de *Escherichia coli* de origen comunitario en un país industrializado.

Este estudio describe la difusión de *E. coli* resistente a las quinolonas en una comunidad indígena muy remota en la selva amazónica Peruana, caracterizada por un nivel de aislamiento muy elevado y un uso muy limitado de antibióticos, que no incluye las quinolonas. Estos hallazgos demuestran que las estrategias de intervención basadas sólo en la restricción de las quinolonas son destinadas a tener un éxito limitado en países de recursos limitados, si no acompañadas de medidas para reducir la diseminación de cepas resistentes mediante la promoción del saneamiento básico y la educación en higiene.
